# Skin and Gut Microbiome in Psoriasis: Gaining Insight Into the Pathophysiology of It and Finding Novel Therapeutic Strategies

**DOI:** 10.3389/fmicb.2020.589726

**Published:** 2020-12-15

**Authors:** Lihui Chen, Jie Li, Wu Zhu, Yehong Kuang, Tao Liu, Wei Zhang, Xiang Chen, Cong Peng

**Affiliations:** ^1^Department of Dermatology, Xiangya Hospital, Central South University, Changsha, China; ^2^Department of Clinical Pharmacology, Xiangya Hospital, Central South University, Changsha, China; ^3^Hunan Key Laboratory of Pharmacogenetics, Institute of Clinical Pharmacology, Central South University, Changsha, China; ^4^Hunan Key Laboratory of Skin Cancer and Psoriasis, Xiangya Hospital, Central South University, Changsha, China; ^5^Engineering Research Center of Applied Technology of Pharmacogenomics, Ministry of Education, Changsha, China; ^6^Central Laboratory, Shenzhen Center for Chronic Disease Control and Prevention, Shenzhen, China

**Keywords:** immunity, microbial interventions, psoriasis, gut microbiota, skin microbiota

## Abstract

Psoriasis affects the health of myriad populations around the world. The pathogenesis is multifactorial, and the exact driving factor remains unclear. This condition arises from the interaction between hyperproliferative keratinocytes and infiltrating immune cells, with poor prognosis and high recurrence. Better clinical treatments remain to be explored. There is much evidence that alterations in the skin and intestinal microbiome play an important role in the pathogenesis of psoriasis, and restoration of the microbiome is a promising preventive and therapeutic strategy for psoriasis. Herein, we have reviewed recent studies on the psoriasis-related microbiome in an attempt to confidently identify the “core” microbiome of psoriasis patients, understand the role of microbiome in the pathogenesis of psoriasis, and explore new therapeutic strategies for psoriasis through microbial intervention.

## Introduction

Over the past two decades, numerous studies have revealed the physiological and metabolic effects of the microbiome on multicellular organisms, with implications for both health and disease. The human microbiome has become an area of utmost interest. Traditional culture-dependent assays have limited the study of the human microbiome and have severely underestimated the diversity of microbial communities in the context of the human body (Tlaskalova-Hogenova et al., 2004). The application of next-generation sequencing, including phylogenetic marker gene-based amplicon studies [e.g., bacterial 16S or fungal internal transcribed spacer (ITS) sequencing] and shotgun metagenomic sequencing, has greatly facilitated research on the human microbiota, allowing us to uncover the mysteries of our second genome, acquired after birth, and to explore the interactions of the microbiota with the host. The internal and external surfaces of many parts in the human body, including the gastrointestinal tract, skin, oral mucosa, vagina and airway, are niches that are colonized by microbes. Among these niches, the colon is the site that most commensal microbes colonize, harboring an estimated 10^14^ bacterial cells (Savage, 1977), followed by the skin, which harbors approximately two orders of magnitude fewer bacterial cells than the colon (Berg, 1996).

The gut, as the anatomical site with the highest distribution of microorganisms, is rich in nutrients and has a pH close to neutral. Some bacteria are not only prevalent across individuals but also are high in abundance, for example, the genus *Bacteroides* and anaerobic cocci, whereas some bacteria are abundant in individual fecal samples but not prevalent among individuals, such as the genus *Clostridium*, members of the genera *Bifidobacterium*, *Eubacterium*, *Lactobacillus*, *Streptococcus*, and some facultative anaerobes like *Escherichia* (Lloyd-Price et al., 2016). In the past, the diversity and functions of gut microbes have been severely underestimated due to the reliance on culture-based microbial research. Compared with the nutrient-rich gut, which is well-suited to microbial growth, skin has a low microbial biomass. This unique ecosystem also harbors specific skin-resident microbes, which are predominated by Gram-positive bacteria and facultatively anaerobic microbes (Grice et al., 2009; Bewick et al., 2019), such as *Actinobacteria*, *Firmicutes*, *Proteobacteria*, and *Bacteroidetes*. The most dominant genera seen on skin include *Corynebacterium*, *Propionibacterium, Streptococcus*, and *Staphylococcus* (Grice and Segre, 2011; Christensen and Bruggemann, 2014; Byrd et al., 2018). The functional and taxonomic compositions of skin-resident microbial communities, characterized by topographical and temporal diversity, are influenced by multiple factors, including lipid content, pH, sweat, sebum secretion and local skin anatomy. Over the last 40 years, much evidence has shown that skin microbes are involved in the development of non-infectious skin diseases, such as acne vulgaris (Holland et al., 1977), rosacea (Thomsen et al., 1980; Till et al., 2000) and psoriasis (Paulino et al., 2006). However, the contribution of the gut microbiome to common skin disorders has not been studied extensively. The recent rapid progression of NGS and bioinformatics technologies has provided access to the composition and genetic functions of the entire gut microbiome and thus enabled systematic investigation of its role in the pathogenesis, flare-up and prognosis of dermatosis.

Psoriasis, a common chronic inflammatory dermatosis, impacts 1–3% of the world’s population. The exact factors that drive psoriasis are not fully understood, but it is considered to be a complicated immune-mediated disease and is affected by both human genetics and environmental factors such as diet, lifestyle and health history. Psoriasis is associated with increased skin inflammation, hyperproliferation of keratinocytes, and overactive IL-17- and IFN-γ-producing T cells and inflammatory dendritic cells (DCs) (Roberson and Bowcock, 2010; Lowes et al., 2014; Afifi et al., 2017). There is no effective cure for this condition. Psoriasis is often commonly accompanied by systematic diseases such as IBDs, psoriatic arthritis, obesity and insulin resistance, which severely influence patients’ quality of life. Strengthening the understanding of metabolic comorbidities associated with psoriasis is of great significance for understanding the pathogenesis of psoriasis and developing novel therapeutic strategies. In particular, the relationship between psoriasis and obesity is bidirectional, with obesity favoring psoriasis and psoriasis predisposing individuals to obesity (Shipman and Millington, 2011; Cooksey et al., 2018; Dauden et al., 2018). Adipokines, synthesized and secreted by adipocytes, which are regulated by the gut microbiota, play an important role in linking the pathophysiology of psoriasis and obesity (Suarez-Zamorano et al., 2015; Kong et al., 2019). Much evidence has shown that the skin and gut microbiota play a role in host homeostasis and immune response, particularly in Th17 cell development (Nemoto et al., 2013; Geem et al., 2014; Cicerone et al., 2015; Bellone et al., 2020). It has been reported that an IBD-like decrease of *Faecalibacterium prausnitzii* together with an increase of *Escherichia coli* in psoriasis (Eppinga et al., 2016). However, there has been no comparative study of the gut microbiota of people in psoriasis and obesity. A lot of research has reported the gut microbes of obese people, characterized by lower level of *F. prausnitzii* than the healthy controls, while the changes in *E. coli* abundance varied among different research (Million et al., 2013; Fernandes et al., 2014; Yasir et al., 2015).

This review attempts to discuss recent insights into skin and gut microbial communities, including their interaction with host metabolism and the immune system, their composition and functional association with human health and psoriasis, and diagnostic or therapeutic approaches targeting these communities, with a focus on examining the evolution of microbiological intervention and reviewing potential microbial biomarkers associated with the occurrence, transmission, flare-up and severity of the disease, in addition to describing the therapeutic potential of modulating microbial compositions and skin immunology.

## Microbiome Alteration: A Potential Pathogenic Factor for Psoriasis

There is a close association between psoriatic attacks and microbiome alterations (dysbiosis), characterized by altered diversity and composition, as well as blooms of opportunistic pathogens. Psoriasis can be provoked or exacerbated by specific pathogens, including bacteria (*Staphylococcus aureus* and *Streptococcus pyogenes*), viruses (human papillomavirus and endogenous retroviruses), and fungi (*Malassezia* and *Candida albicans*) (Fry and Baker, 2007; Wang and Jin, 2018). Malassezia, the most common fungal species present on normal human skin, show lower abundance in comparison with the healthy controls (Fernandes et al., 2014; Takemoto et al., 2015). Candida species are prevalent on the skin in psoriasis lesions and in the feces of patients with psoriasis (Ovcina-Kurtovic et al., 2016), while interleukin-17, overexpressed in patients with psoriasis, can protect against infections, especially those due to *Candida* spp. (Lonnberg et al., 2014; Saunte et al., 2017), suggesting that the induced overactive Th17 response is a potential pathogenic factor of psoriasis. We need to identify which comes first: abnormal colonization by *C. albicans* or psoriasis, that is, the chicken or the egg. If the former occurs first, specific antibiotics can be used to eliminate the abnormal colonization by the pathogen to prevent the occurrence or development of the disease. *S. aureus*, another common pathogen, may also be a pathogenic factor of psoriasis, given the increase in Th17 polarization and exacerbated cutaneous inflammation during early colonization of newborn mouse skin (Chang et al., 2018).

## Skin Microbiome and Psoriasis

### Cutaneous Homeostasis: A Result of the Interaction Between Skin-Resident Commensals and Cutaneous Immune Cells

Diverse body sites in healthy humans representing distinct skin niches are generally divided into three microenvironment types: dry sites, moist sites and sebaceous sites. Microbial features and disease susceptibility vary across different skin microenvironments (Grice et al., 2009; Belkaid and Segre, 2014; Byrd et al., 2018). Microbial communities colonizing the skin, cells resident in the epidermis and dermis, such as keratinocytes and fibroblasts, as well as skin immune cells together shape the cutaneous immune system. Commensal microorganisms that colonize the cutaneous surface have an effect on maintaining human skin homeostasis, in addition to educating and priming the cutaneous immune response in the state of pathogen invasion. Commensals can directly inhibit pathogen growth by capturing nutrients and space in the skin and producing antimicrobial peptides (AMPs) or bactericidal compounds; for example, colonization by the commensal *Staphylococcus epidermidis* inhibits exposure to the pathogen *S. aureus* during early life, preventing inflammatory diseases (Kennedy et al., 2017; Nakatsuji et al., 2017; Parlet et al., 2019; Gonzalez et al., 2020). Moreover, myriad immune cells are induced by commensals to provide protection against pathogens. *Corynebacterium accolens* dominates the skin microbial communities, or its membranous derivative mycolic acid protects against the common microbial pathogens *S. epidermidis* and *C. albicans* through the expansion of IL-17-producing dermal γδ T cells (Ridaura et al., 2018). Interleukin-17A (IL-17A)-producing T cells [CD8+ (T_C_17) and CD4+ (T_H_17)], which are commensal-specific T cells, can be induced upon encountering commensalism under homeostasis and then function as lasting tissue-resident memory cells. Harrison et al. showed that commensal-specific type T cells can mediate antimicrobial defense in the steady state but rapidly adapt to injury and promote tissue repair (Harrison et al., 2019). Colonization by *S. epidermidis* also shapes the skin’s T cell network. Monoassociation of *S. epidermidis* has the ability to strengthen cutaneous CD8+ T cells to produce IFNγ and IL-17 effector functions that protect against the skin pathogens *Leishmania* and *C. albicans* (Naik et al., 2012). In addition, cutaneous bacteria can be involved in immune tolerance to commensals and their metabolites. Exposure to *S. epidermidis* in early life contributes to skin homeostasis by triggering an influx of CD4+ T regulatory cells, thus mediating immune tolerance to commensal antigens (Ags) (Scharschmidt et al., 2015). Moreover, skin microbes can also be involved in the development and maturation of cutaneous immune cells. For example, the maturation of mast cells in mice, leaving the bone marrow and eventually settling in the skin, is dependent on skin microbes that promote stem cell factor (SCF) production in KCs (Wang et al., 2017). Another study found that the recruitment of MCs and SCF expression in mouse skin were regulated by the hairless-skin microorganisms (Wu C. C. et al., 2019). Early colonization by microorganisms is critical to the development of the human immune system and has a long-term impact on human health. Mucosal-associated invariant T (MAIT) cells, controlling tissue repair and homeostasis, are imprinted by microbes and induced in response to riboflavin-synthesizing commensals during a specific early life window (Constantinides et al., 2019). In turn, cutaneous immune cells such as DCs, mast cells, natural killer cells, macrophages and various T cells, involved in both innate and adaptive immune responses, shape resident microbial communities in skin. For instance, skin-resident innate lymphoid cells (ILCs) can tune the microbial commensal equilibrium to control the homeostasis of sebaceous glands. The absence of ILCs leads to sebaceous hyperplasia, accompanied by increased production of antibacterial lipids and the restriction of commensals of Gram-positive bacterial communities (Kobayashi et al., 2019). In short, skin commensals maintain cutaneous homeostasis by balancing immune defense against pathogens and immune tolerance to commensal antigens.

Skin, both a physical barrier and an immunologic barrier to external threats, is tightly controlled by its resident microbiota (Chen Y. E. et al., 2018). Psoriasis as an inflammatory skin disease showed decreased barrier function, but the underlying mechanisms remain unclear (Madison, 2003). There is much evidence that psoriatic fares are associated with skin microbiota alterations and the microbiota is a major player in the etiology of this condition (Fahlen et al., 2012; Alekseyenko et al., 2013; Zakostelska et al., 2016). Repeated infection of the skin surface is also closely associated with the development and progression of psoriasis (Alekseyenko et al., 2013; Hurabielle et al., 2020). Disruption of epidermal barrier integrity due to disturbance of the composition and function of the resident microbial community in the skin is significantly related to psoriasis, including some skin fungal flora (*Candida albicans*) and bacterial flora (*Corynebacterium* species), which might induce the accumulation of Th17 cells (Zielinski et al., 2012; Tauch et al., 2016). Skin resident microbiota and epidermal immune cells work together to maintain the skin barrier structure and function, and this structure might be damaged under pathological conditions, thus contributing to inflammatory responses. In most cases, however, it is not clear that abnormal immunity to the microorganisms is a bystander to the inflammatory process or a driver and/or magnifier of the disease states, which remains under investigation.

### Skin Dysbiosis in Psoriasis

Microbiome perturbations are associated with several immune-mediated dermatoses, such as psoriasis, atopic dermatitis and acne vulgaris. Each disease has distinct skin microbiological characteristics. Many studies have shown that the composition and function of the skin microbiome vary between patients with psoriasis and healthy controls and psoriatic flares were associated with skin microbiome alterations; however, the lack of standardized sampling and profiling protocols also leads to conflicting results. Here, we review recent advances in the study of the close, non-causal association between microbial alteration and psoriasis, attempting to gain insight into the core microbial composition associated with psoriasis and to assess the potential diagnostic value of dysbiosis.

A search performed on October 2020 in the databases Pubmed and Web of Science, using the terms: “skin microbiota,” “skin microbiome,” “psoriasis,” and “humans.” Observational human studies and clinical trials that evaluated the skin microbiota or microbiome composition in psoriasis patients and in those of healthy controls using cultivation-independent methods, written in English, were included. Studies with animal models; studies not compared with healthy controls or unaffected skin regions of the same individual; and studies with a high risk of bias (poor quality), were excluded. Fifty three studies have been found in the databases Pubmed and Web of Science after the removal of duplicates and screening of the title and abstract, and the full texts of only 24 articles were read after the removal of reviews, systematic reviews. Here we describe the whole selected studies in which skin microbes of psoriatic in different disease states were compared and analyzed. Studies compared with healthy controls by rRNA community profiling, are included and described in Table 1.

**TABLE 1:** Reported skin dysbiosis in psoriasis.

Sample size	Amplification area	Skin dysbiosis in psoriasis	Sampling methods	References
28 psoriasis patients and 26 healthy subjects	16S rRNA V1–V3 variable region	At the phylum level, *Actinobacteria↓ Proteobacteria↑* At the genus level, *Propionibacterium, Ethanoligenens*, and *Macrococcus↓ Pseudomonas↑* At the species level, *Staphylococcus epidermidis, Propionibacterium acnes*, and *Propionibacterium granulosum↓ Staphylococcus aureus and Staphylococcus pettenkoferi*↑	Swab	Chang et al., 2018
54 patients with psoriasis and 37 healthy controls	16S rRNA V1–V3 variable region	At the phylum level, *Proteobacteria↓ Actinobacteria* and *Firmicutes*↑	Swab	Alekseyenko et al., 2013
10 patients with psoriasis and 12 healthy controls	16S rRNA V3–V4 variable region	At the phylum level, *Proteobacteria*↑ *Firmicutes* and *Actinobacteria↓* At the genus level, *Streptococci* and *Propionibacteria*↓ *Staphylococci*↑	Biopsy	Fahlen et al., 2012
3 male first cousins	V2–4–8 and V3–6, 7–9 regions of the bacterial 16S rRNA genes	At family level, *Streptococcaceae*, *Rhodobacteraceae*, *Campylobacteraceae*, and *Moraxellaceae* At the phylum level, *Firmicutes↓ Proteobacteria↑* At the genus level, *Staphylococcaceae* and *Propionibacteriaceae↓* At the species level, *Propionibacterium acnes*↓	Swab	Drago et al., 2016
82 patients with atopic dermatitis, 119 patients with psoriasis and 115 healthy controls	16S rRNA V1-V4 variable region	At the species level, *Corynebacterium simulans*, *Corynebacterium kroppenstedtii*, *Finegoldia* and *Neisseriaceae↑ Lactobacilli, Burkholderia* spp. and *Propionibacterium acnes*↓	Swab	Fyhrquist et al., 2019
27 patients with psoriasis and 19 healthy controls	16S rRNA V3–V4 variable region	At the phylum level, Deinococcus-Thermus↓ At the genus level, *Propionibacterium↓ Corynebacterium*↑	Swab	Quan et al., 2020
50 patients with plaque-type psoriasis and 77 healthy controls	16S rRNA V3-V4 variable region	At the phylum level, *Firmicutes* and *Protebacteria↑ Fusobacteria* and *Cyanobacteria*↓	swab	Assarsson et al., 2020

Based on sequencing of the 16S rRNA V1-V3 variable region, Chang et al. (2018) revealed that different disease states (healthy, psoriatic lesional, and psoriatic non-lesional) can be discriminated by the taxonomic composition at the phylum and genus levels. Strikingly, the genera *Staphylococcus* as a whole was not significantly discriminated with any skin condition, while the relative abundance of the *Staphylococcus* species across all samples is associated with different disease states (see Table 1), suggesting that the dynamic inter-microbe relationship between different *Staphylococcus* species might lead to the various microbial communities associated with healthy and psoriatic skin. The genus *Pseudomonas*, enriched with psoriatic skin, includes many opportunistic pathogens and contributes to the treatment response of psoriasis patients treated with narrowband ultraviolent B (UVB) (Assarsson et al., 2018). For example, the most prominent bacteria of Gram-negative toe-web infection, *Pseudomonas aeruginosa*, is closely associated with the incidence of psoriasis (Goiset et al., 2019). Chang et al. (2018) also found that microbial communities in psoriatic lesions tended to display higher alpha diversity and greater heterogeneity but lower stability than healthy skin microbial communities. In contrast, Alekseyenko et al. (2013) reported that the microbiome of psoriatic lesions displayed decreased taxonomic diversity and was enriched with *Firmicutes* and *Actinobacteria*. What’s more, two taxa were found to be correlated best with psoriasis status, *Acidobacteria Gp4* and *Schlegelella*, which were either not found in the HMP subjects (Gp4), or found only rarely. The combination of these two taxa might serve as potential markers for distinguishing skin from different disease states. This study ensures the matching of skin sites among different groups during sampling to avoid the influence of different skin sites and skin types on the skin microbial community. Gao et al. (2008) also showed that the *Firmicutes* phylum was overrepresented, while the *Actinobacteria* phylum and *Propionibacterium* species were underrepresented, in psoriatic lesions by applying RT-PCR. Fyhrquist et al. (2019) applied a larger-scale sample to profile the skin microbiota associated with atopic dermatitis, psoriasis and healthy volunteers and found that reduced abundance of members of *Corynabacterium* might play a regulatory role in psoriasis. *Corynebacterium* species of the human microbiome have often been assigned as opportunistic pathogens and *Corynebacterium kroppenstedtii*, showing a higher level of abundance in skin microbiota of psoriasis patients than healthy subjects, has occasionally been related to human infections, mainly granulomatous mastitis and breast abscesses (Tauch et al., 2016). Quan et al. (2020) also revealed that the higher level of *Corynebacterium* and the lower level of *Cutibacterium* were closely associated with psoriatic lesions. Based on sequencing of the 16S rRNA V3-V4 variable region, Fahlen et al. (2012) observed a lower diversity in psoriatic lesions compared to the control group. Both *Staphylococci* and *Propionibacterium* were present at lower levels, while *Proteobacteria* was present at higher levels, in psoriatic subjects than in controls. It is worth mentioning that the region of amplification and the method of sampling used in this study are different from others, which might contribute to the distinct results. Drago et al. (2016) found an increase in *Proteobacteria* abundance and a decrease in the abundances of *Streptococcaceae*, *Rhodobacteraceae*, *Campylobacteraceae*, and *Moraxellaceae* as well as *Firmicutes* in psoriatic subjects compared to healthy controls. The sample size of the study was so small that the reliability of the results remains to be further confirmed, despite strict dietary controls on the included individuals. Another study, applying shotgun metagenomics to profile the microbiome of psoriatic and unaffected skin from 28 patients with plaque psoriasis, found that the microbial communities of psoriatic and unaffected sites showed little difference at the species level while strain heterogeneity colonization and functional variability were revealed, which showed that higher-resolution analyses would be needed to clarify the pathogenesis of psoriasis and identify new therapeutic targets for it (Tett et al., 2017). Paulino et al. (2006) revealed that *Malassezia* microbiota was host-specific and relatively stable over time and there was no significant difference between samples from healthy skin and psoriatic lesions, using multiplex real-time PCR.

After analyzing most of the studies on the relationship between skin microbes and psoriasis, we found that the variation trends of the *Firmicutes*, *Actinobacteria*, and *Proteobacteria* abundances in various studies were highly controversial. In addition, there was no convincing conclusion regarding whether the diversity of the microbial community on psoriatic lesional skin lower than that on healthy skin. What is certain, however, is that increased *S. aureus* abundance and decreased *S. epidermis* abundance were observed in psoriatic lesions (Ng et al., 2017; Liu S. H. et al., 2019). The reports mentioned above are qualitative in nature, and the loss of quantitative definition directly limits insights into what features are reproducible across studies. What makes the results so different is that skin microbes are affected by a variety of host and environmental factors, such as daily hygiene regimens, use of cosmetic products, exposure to antimicrobials, friction, climate, and UV irradiation (Babeluk et al., 2014; Lee H. J. et al., 2018; Burns et al., 2019; McBain et al., 2019). Therefore, these confounders must be strictly controlled during study so that the results across samples and studies can be comparable. In addition, distinct skin sites and skin types, the selection of primer to amplify regions for sequencing and sampling methods including swab, scrape and punch biopsy, contribute to differences in experimental results, particularly on the diversity of the microbial community (Grice et al., 2008; Claesson et al., 2010; Alekseyenko et al., 2013; Castelino et al., 2017; Chang et al., 2018; Stehlikova et al., 2019).

The reports mentioned above are qualitative in nature, and the loss of quantitative definition directly limits insights into what features are reproducible across studies. To gain insight into the characteristic changes in psoriatic lesions, studies with large sample sizes, standardized protocols and sufficient sequencing depth are required. Alternatively, a scientific meta-analysis of publicly available data from studies on the relationship between skin microbes and psoriasis can help address discrepancies in an unbiased manner, and after devising valid statistical methods to exclude experimental and human confounding factors, the microbial alteration associated with psoriasis may be reliably identified.

## Gut Microbiome and Psoriasis

### The Role of the Gut Microbiota in Maintaining a Healthy Gut Ecosystem

Gut microbes and their metabolites contribute to host health by digesting food and maintaining immune system homeostasis. The gut flora helps digest and break down complex polysaccharides and is crucial for the production of some important nutrients, such as vitamin K and B-group vitamin (Hill, 1997; Karasov et al., 2011; LeBlanc et al., 2013; Heintz-Buschart and Wilmes, 2018). The intestinal microbiota plays a vital role in maintaining epithelial barrier integrity and forming a mucosal immune system to protect against invasion by exogenous pathogens and for balancing host defense and tolerance of dietary and environmental antigens through bacterial metabolites and components. The gut microbiota and its metabolites, intestinal epithelial cells (IECs) and immune cells participate in the maintenance of a healthy gut ecosystem. Indeed, the gut microbiota is a non-self entity and would be eliminated if the immune cells residing in the intestinal mucosa recognized it. The intestinal epithelium serves as a barrier to isolate immune cells residing in the intestinal mucosa from the microbiota present in the intestinal lumen but allows microbial metabolism to gain access to and interact with host cells and thus regulate immune responses.

The interactions between the microbiota and the host are mediated mainly through bacterial components and microbial metabolites. The gut microbiota contributes to innate immune responses via interactions with Toll-like receptors (TLRs) and pattern recognition receptors (PRRs) on the surface of innate immune cells (Rooks and Garrett, 2016; Weaver et al., 2019). In addition, gut microbes participate in the adaptive immune response by inducing the secretion of immunoglobulin A (IgA) and affecting effector T cells (Th1, Th2, Th17), regulatory T cells and Tfh cell member and function (Bunker et al., 2015; Honda and Littman, 2016; Teng et al., 2016; Yissachar et al., 2017). For example, spore-forming bacteria, such as *Clostridia*, can induce the colonic Tregs, rebalance Th1/Th2/Th17 cells and change to a less proinflammatory immunological milieu in the gut (Jia et al., 2017). Bacteroides fragilis and segmented filamentous bacteria (SFB) have been reported to induce intestinal Tregs and Th17 cell differentiation, respectively, thus affecting the host resistance against infections and promote systemic autoimmunity (Ivanov et al., 2009; Round and Mazmanian, 2010; Wu et al., 2010).

Microbiota-produced metabolites in the intestine, such as short-chain fatty acids (SCFAs), secondary bile acids and tryptophan, balance the activation and suppression of the immune system and affect multiple organs throughout the body. For example, SCFAs, especially butyrate, inhibit immune responses by inhibiting the proliferation, migration, adhesion, and cytokine production of inflammatory cells (Meijer et al., 2010; Schwarz et al., 2017). In addition, SCFAs inhibit histone deacetylase and inactivate NF-κB signaling pathways to tune the activation and apoptosis of immune cells (Meijer et al., 2010; Alekseyenko et al., 2013; Schwarz et al., 2017). There is much evidence of the important role of microbial metabolites of tryptophan in balancing host defense and microbiota homeostasis. Indole-3-aldehyde (IAld), an indole derivative of tryptophan catabolism by the gut microbiota, tunes mucosal reactivity through IL-22 and protects against colonization by *C. albicans*. The microbial metabolites of tryptophan also affect central nervous system inflammation and astrocyte activity. Moreover, IAld, a tryptophan metabolite derived from the skin microbiota, attenuates inflammation in patients with atopic dermatitis through the aryl hydrocarbon receptor. None of these phenomena would have been observed in germ-free mice (Zelante et al., 2013; Rothhammer et al., 2016). The upregulated tryptophan metabolism pathway is also observed in patients with psoriasis, while the role of microbes in the pathway remains to be explored (Harden et al., 2016). Other microbial metabolites, such as polysaccharide A and retinoic acid produced by commensal *Clostridia*, *Faecalibacterium prausnitzii*, and *Bacteroides fragilis*, induce the accumulation of regulatory T (Treg) cells, which suppress inflammation (Forbes et al., 2016).

**FIGURE 1:**
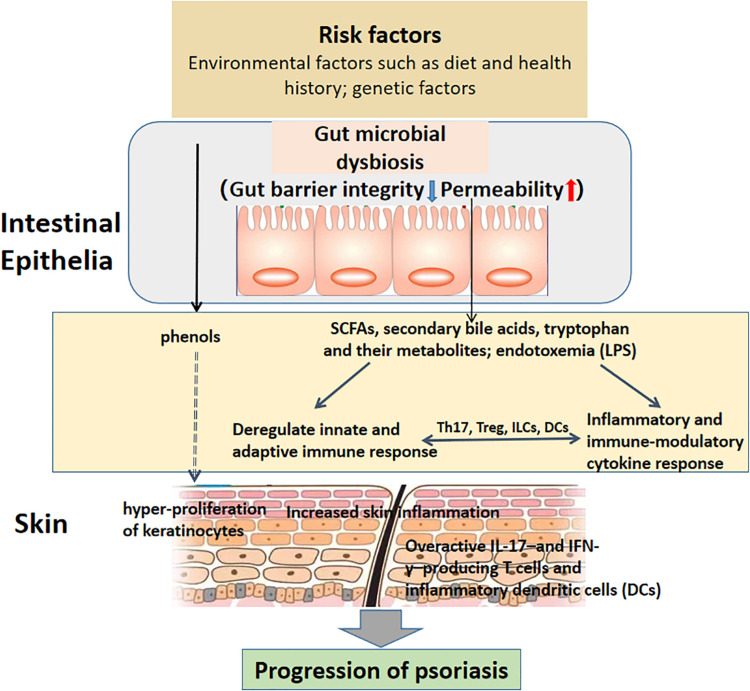
The putative relationship between gut dysbiosis and psoriasis onset and progression. Intestinal barrier function is maintained by group 3 innate lymphoid cells (ILC3s) and IL-17- and IL-22-producing T helper 17 (Th17) cells, which modulate antimicrobial peptide secretion by intestinal epithelial cells (IECs) and IgA production in the gut (Hirota et al., 2013; Kruglov et al., 2013). Moreover, dendritic cells (DCs) participate in microbiota sensing via the Mincle-Syk axis to regulate IL-17 and IL-22 production and promote intestinal barrier integrity (Martinez-Lopez et al., 2019). Host- and microbiota-derived factors induce gut microbe dysbiosis, including disruption of gut barrier integrity and increased permeability, as well as alterations in microbial metabolites such as SCFAs, secondary bile acids, tryptophan, lipopolysaccharides (LPSs) and phenols, thus disturbing immune homeostasis via a low-grade chronic inflammatory process. For example, the intestinal microbiota promotes psoriasis-like skin inflammation by enhancing the Th17 response, and regulatory T cell (Treg) levels decrease in psoriasis patients, leading to an imbalance between effector T cells and suppressor T cells (Zakostelska et al., 2016; Komine, 2020). Th17 cell differentiation requires IL-6 and transforming growth factor-β (TGF-β) from DCs in an antigen-dependent manner (Ivanov et al., 2006; Persson et al., 2013). In contrast, activation of ILC3s requires the release of IL-23 by myeloid cells to produce IL-22 and/or IL-17, which is antigen dependent (Satpathy et al., 2013; Longman et al., 2014). Increased numbers of ILC3s exist in the circulating blood of psoriatic arthritis patients, as well as the lesional and non-lesional skin of psoriasis patients (Soare et al., 2018). Phenols, as metabolites of aromatic amino acids produced by gut bacteria and regarded as bioactive toxins and serum biomarkers of a disturbed gut environment, have the ability to influence keratinocyte differentiation in the skin, but the underlying mechanism remains to be explored (Miyazaki et al., 2014). The involvement of immune cells and their active factors and intestinal microorganisms and their metabolites promotes the progression of psoriasis.

The gut microbiota has a regulatory effect on systemic immunity, causing the functioning and dysfunction of distant organ systems. Dysbiosis, involving alteration in the composition and function of microbial communication, may result in increased gut barrier permeability, which contributes to immune activation by translocation of microbial antigens and their metabolites into the blood circulation (Wells et al., 2017). Destroyed intestinal integrity and increased gut permeability, consequences of the systemic inflammation fueled by gut dysbiosis, has been implicated as a factor contributing to local and systemic immune response, and the pathogenesis of psoriasis (Lin and Zhang, 2017). The skin has a particularly complicated connection with the gut, but the underlying mechanism is still completely understood, and this phenomenon is at least partially attributed to the disruption of the intestinal barrier (Kim et al., 2014; Kosiewicz et al., 2014; Camilleri, 2019). Oral administration of *S. aureus* and *Streptococcus danieliae*, which are abundant in the inflammatory skin mouse model, exacerbated skin inflammation associated with imiquimod-induced psoriasis-like dermatitis with elevated levels of TNF-α, IL-17A, IL-17F and IL-22, also suggesting a potential role of gut dysbiosis in the development of psoriasis (Okada et al., 2020). What’s more, Sikora et al. (2018) assessed non-invasive markers of intestinal barrier integrity in psoriasis patients, including concentrations of claudin-3, intestinal fatty acid binding protein (I-FABP) in the blood in psoriasis patients and healthy controls. They found that psoriasis patients had more elevated concentration of plasma claudin-3 and I-FABP, supporting the hypothesis that dysfunction of the intestinal barrier in psoriasis disturbs the homeostatic equilibrium between the microbiota and immune system. In another study conducted by Sikora confirmed that I-FABP is associated with severity of psoriasis (Sikora et al., 2019). The putative relationship between gut dysbiosis and psoriasis development and progression have been shown in Figure 1.

### Intestinal Dysbiosis in Psoriasis

The intestinal microbiota contributes to the balance between Th17 effector cells and their counterpart regulatory T cells (van Beelen et al., 2007), and Zakostelska et al. (2016) showed that imiquimod induced milder psoriasis-like skin inflammation in germ-free mice than in conventional mice by enhancing the Th17 response, suggesting that gut dysbiosis acts as a potential pathogenic factor for psoriasis.

Studies on the correlation between intestinal microorganisms and psoriasis are limited and surged in recent years, and we searched in the databases Pubmed and Web of Science on October 2020, using the terms: “gut microbiota,” “gut microbiome,” “psoriasis,” and “humans.” Observational human studies and clinical trials that evaluated the gut microbiota composition in psoriasis patients and in those of healthy controls using cultivation-independent methods, and written in English (see Table 2), were discussed below.

**TABLE 2:** Reported intestinal dysbiosis in psoriasis.

Sample size	Amplification area	Intestinal dysbiosis in psoriasis	Function prediction	References
32 psoriatic patients and 64 healthy controls	V3–V4 region of bacterial 16S rRNA genes	At the family level, *Ruminococcaceae* and *Lachnospiraceae↑ Bacteroidaceae* and *Prevotellaceae↓* At the phylum level, *Firmicutes↑ Bacteroidetes↓* At the genus level, *Ruminococcus and Megasphaera*↑	Functional genes and metabolic pathways involving bacterial chemotaxis and carbohydrate transport were predicted to be over-represented; genes related to cobalamin and iron transport were predicted to be underrepresented.	Chen Y. J. et al., 2018
35 psoriatic patients and 27 healthy controls	V4 and V5 regions of the bacterial 16S rRNA genes	At the phylum level, *Firmicutes, Proteobacteria*, and *Actinobacteria↑ Bacteroidetes↓* At the genus level, *Bacillus, Bacteroides, Sutterella, Lactococcus, Lachnospiraceae_UCG004, Lachnospira, Mitochondria_norank, Cyanobacteria_norank*, and *Parabacteroides↑ Thermus, Streptococcus, Rothia, Granulicatella, Gordonibacter, Allobaculum*, and *Carnobacterium↓* At the genus level, *Bacteroides*↓ *Faecalibacterium, Akkermansia* and *Ruminococcus*↑	/	Huang et al., 2019
52 psoriatic patients and 300 healthy controls extracted from the Human Microbiome Project	Broad-range PCR amplification of the bacterial 16s rRNA gene conserved region	Genus *Bacteroides*↓ *Akkermansia spp.*↑	/	Codoner et al., 2018
24 psoriatic patients and 22 healthy controls	V4 region of the bacterial 16S rRNA genes	At the phylum level, *Firmicutes* and *Actinobacteria↑ Bacteroidetes* and *Proteobacteria↓* At the genus level, *Blautia↑ Prevotella↓* At the species level, *Ruminoccocus gnavus, Dorea formicigenerans*, and *Collinsella aerofaciens Prevotella copri*↓	Genes encoding energy metabolism and involved in glutathione synthesis were predicted to be underrepresented; genes encoding butyrate kinase and phosphate butyryltransferase were predicted to be underrepresented; pathways related to lipopolysaccharide (LPS) biosynthesis were predicted to be over-represented.	Shapiro et al., 2019
16 patients with psoriatic arthritis, 15 patients with psoriasis of the skin and 17 healthy, matched control subjects	V1–V2 region of bacterial 16S rRNA genes	At the genus level, *Parabacteroides, Coprobacillus*, unclassified *Ruminococcaceae*, unclassified *Lachnospiraceae*↓	Decreased quantities of MCFAs	Scher et al., 2015
19 patients with psoriasis and 20 healthy controls	V2–V3 region of bacterial 16S rRNA genes	At the family level, *Bifidobacteriaceae, Coriobacteriaceae, Lachnospiraceae, Clostridiales Family XIII, Eggerthellaceae, Peptostreptococcaceae, Ruminococcaceae* and *Erysipelotrichaceae*↑ *Bacteroidaceae, Barnesiellaceae, Prevotellaceae, Tannerellaceae, Burkholderiaceae, Rikenellaceae, Lactobacillaceae, Streptococcaceae, Desulfovibrionaceae, Veillonellaceae, Marinifilaceae, Victivallaceae*↓ and *Pasteurellaceae* At the phylum level, *Actinobacteria* and *Firmicutes*↑ *Bacteroidetes* and *Proteobacteria*↓	/	Hidalgo-Cantabrana et al., 2019

Many studies have shown that the gut microbiota associated with psoriasis patients significantly differs from that in healthy subjects. Tan et al. (2018) showed that the abundance of *Akkermansia muciniphila* was markedly reduced in patients with psoriasis. Chen Y. J. et al. (2018) revealed an increased abundance of the phylum *Firmicutes*, from which *Ruminococcus* and *Megasphaera* were the top two genera of discriminant abundance, and a decreased abundance of the phylum *Bacteroidetes* was observed in psoriasis patients, which might be associated with suppressed cobalamin and iron transport. Obesity and the drug effect were considered as confounders in this study and a significant difference in bacterial composition between psoriasis and controls was found in those with BMI values less than 25 but not among obese subject (BMI ≥ 25). Huang et al. (2019) confirmed that *Bacteroidia* was a key factor contributing to the dysbiosis of microbiota in psoriasis patients, while the phylum *Firmicutes* was the key factor contributing to the distribution of the microbiota in healthy subjects. This finding was supported by a previous study reporting that the ratio of *Firmicutes* and *Bacteroidetes* was perturbed in psoriasis patients. There were no significant differences in intestinal microbial composition observed in psoriasis with distinct severity. Codoner et al. (2018) found a different psoriatic gut microbiome (with high diversity), an increased presence of *Faecalibacterium* and a decrease in the abundance of *Bacteroides*, as well as higher levels of the genera *Akkermansia* and *Ruminococcus*, in psoriatic patients. Three hundred healthy controls extracted from the Human Microbiome Project were involved in this study, which made it impossible to ensure a good match of other confounders (age, gender, dietary habit and BMI index) between healthy controls and psoriatic patients, thus perturbing the results. Shapiro et al. (2019) demonstrated a significant increase in the phyla *Firmicutes* and *Actinobacteria* phyla in psoriatic patients compared with matched controls, and psoriatic patients showed significant increases in the abundances of species such as *Ruminoccocus gnavus*, *Dorea formicigenerans*, and *Collinsella aerofaciens*, while species such as *Prevotella copri* and *Parabacteroides distasonis* were significantly depleted in psoriasis patients compared with controls. Small sample size, insufficient sequencing depth, possible contamination in the experimental process, and various factors affecting the microbial composition of feces, such as diet, in addition to a lack of quantitative definition, make the results unreliable, and better methods remain to be explored to provide access to the relationship between psoriasis and strain-level intestinal bacteria so that psoriasis can be diagnosed and treated with microbial interventions. Moreover, the intestinal microorganisms of psoriatic patients can be compared with those of non-psoriatic patients with obesity, IBD, irritable bowel syndrome (IBS) or other diseases closely related to psoriasis to further understand the role of microorganisms in the interaction of obesity (IBD, IBS) and psoriasis and propose new microbiota-related treatments for the two conditions in the future.

## Modulation of the Gut Microbiota for Prevention and Treatment: From Biology to Clinic

Given the gut microbial contribution to inflammatory disease and the immune system, there exists an opportunity to intentionally modulate the microbiome for therapeutic purposes. The shaping of gut microbial composition and function depends largely on environmental factors, particularly diet, rather than human genetics. Restoration of the gut microbiome is a promising preventive and therapeutic strategy in a number of clinical conditions.

### Fecal Microorganism Transplantation (FMT)

Myriad studies have reported alterations in the composition and function of the intestinal microbial community and improvements in disease through long-term oral supplementation with food, prescription medicine or traditional herbal products and explored the underlying mechanism (Sung et al., 2017; Lee H. et al., 2018; Sharma et al., 2018; Ejtahed et al., 2019; Liu M. T. et al., 2019; Sheng et al., 2019; Wu T. R. et al., 2019; Xu et al., 2019; Zhang et al., 2019; Wang et al., 2020). Additionally, there is much evidence from both mouse and human studies that the recipient’s microbial community can be gradually rectified to normalize the microbial composition and function by transplanting fecal microorganisms associated with a “healthy” state, which thus have a therapeutic effect, especially in the treatment of recurrent *Clostridium difficile* infection (CDI) and in the improvement of obesity. A 36-year-old male patient diagnosed with severe psoriasis for 10 years and IBS for 15 years was subjected to FMT twice via both upper endoscopy and colonoscopy with a 5-week interval. After the second FMT treatment for 5 weeks, the two conditions improved greatly (Lai et al., 2018; Mullish et al., 2018; Sun et al., 2018; Krajicek et al., 2019; Yin et al., 2019). Although FMT is potentially effective in many microbiota-related disorders, helping establish trans-kingdom equilibrium between gut fungi, viruses and bacteria to promote the restoration of microbial homeostasis (McSweeney et al., 2020), the effect of FMT depends on several factors, including the bacterial load and microbial composition and function of the recipient and the donor, the physiological and genetic factors related to the recipient and the donor, and FMT preparation and route of administration (Fischer et al., 2016; Vermeire et al., 2016; Duvallet et al., 2017; Ianiro et al., 2017; Allegretti et al., 2019, 2020; Ding et al., 2019; Paramsothy et al., 2019; Ramai et al., 2019; Wilson et al., 2019; de Groot et al., 2020; McSweeney et al., 2020).

### Probiotics and Prebiotics With Beneficial Effects in Psoriasis

Fecal microorganism transplantation is not a one-size-fits-all approach, and studies are needed to identify microbial active components that have specific effects in patients with different diseases. This promotes the therapeutic view of regulating systemic immunity by manipulating the intestinal microbiota community either through stimulating bacterial growth via supplementation with prebiotics (non-viable bacterial components and metabolites) or through expansion via administration of probiotics (live beneficial gut bacteria) and synbiotics (combinations of probiotics and prebiotics) (see Table 3). Probiotic supplementation has a promising potential role in the prevention and management of various skin conditions. Chen et al. (2017) revealed that oral administration of *Lactobacillus pentosus* GMNL-77, a potential probiotic strain, significantly decreased erythaematous scaling lesions in imiquimod-treated mice with epidermal hyperplasia and psoriasis-like skin inflammation, with decreased tumor necrosis factor-alpha (TNF-α), interleukin (IL)-6, and IL-23/IL-17A axis-associated cytokine (IL-23, IL-17A/F, and IL-22) levels in the skin and reduced IL-17- and IL-22-producing CD4+ T cells in the spleen. Ethanol extract (SEL001), isolated from the potent probiotic strain *Lactobacillus sakei* proBio-65, has a protective effect on imiquimod-treated psoriasis-like skin inflammation in a mouse model, with decreased gene expression levels of IL-19, IL-17A, and IL-23 (Rather et al., 2018). In a documented case of severe pustular psoriasis that did not respond to steroids, dapathon, and methotrexate, clinical improvement was observed within 2 weeks after initiation of *Lactobacillus sporogenes* supplementation three times a day, and almost complete resolution was observed at 4 weeks (Sikora et al., 2019). In a separate placebo-controlled study of psoriasis patients, *Bifidobacterium infantis* 35624 supplementation resulted in significantly reduced plasma levels of TNF-α, IL-6 and C-reactive protein (CRP) in the probiotic-treated group (Groeger et al., 2013).

**TABLE 3:** Evidence of the beneficial effects of probiotics in psoriasis.

Patients or mice	Probiotic intervention	Clinical outcome	References
Imiquimod-treated mice with psoriasis-like skin inflammation	Oral administration of *Lactobacillus pentosus* GMNL-77	(1) decreased erythaematous scaling lesions; (2) decreased TNF-α, IL-6, IL-23, IL-17A/F, and IL-22 levels in the skin; (3) reduced IL-17- and IL-22-producing CD4+ T cells in the spleen	Chen et al., 2017
Same as above	Ethanol extract (SEL001)	Decreased IL-19, IL-17A, and IL-23	Rather et al., 2018
A 47-year-old female who presented with crops of pustules all over her body for 20 days prior	Oral administration of *Lactobacillus sporogenes*, one sachet thrice daily	In 15 days, the fever subsided, lesions started involuting and no new lesions appeared	Vijayashankar and Raghunath, 2012
26 psoriasis patients in three separate randomized, double-blind, placebo-controlled interventions	*Bifidobacterium infantis* 35624	Decreased TNF-α, IL-6 and C-reactive protein (CRP) levels	Groeger et al., 2013
Ninety 18–70-year-old adults with plaque psoriasis	*Bifidobacterium longum* CECT 7347, *B. lactis* CECT 8145 and *Lactobacillus rhamnosus* CECT 8361 with a total of 1*10^9 colony-forming units per capsule	After 12 weeks, more patients in the probiotic group compared to the placebo group showed a reduction in the psoriasis area and severity index of up to 75%	Navarro-Lopez et al., 2019

### Potential Probiotics and Prebiotics for Psoriasis Treatment

Some probiotics have been shown to ameliorate skin inflammation by modulating immune responses in the host, but there is no evidence of their potential role in treating psoriasis (see Table 4). Oral administration of poly-γ-glutamate, a natural product of a few Gram-positive bacteria, including *Staphylococcus* and *Bacillus* species, ameliorates AD-like dermatitis in Nc/Nga mice by suppressing the Th2-biased immune response and production of IL-17A, which may indicate it to be a desirable prebiotic for treating overactive Th17 cells involved in psoriasis (Lee et al., 2014). Oral supplementation with milk fermented with *Lactobacillus casei* or administration of *L. casei* alone can decrease skin inflammation by modulating the pool size of cytotoxic CD8+ T cells (Chapat et al., 2004). Further research revealed that *L. casei* DN-114 001 efficiently ameliorated T cell-mediated skin inflammation via the modulation of cytotoxic CD8+ T cells and the participation of CD4+ Treg cells (Hacini-Rachinel et al., 2009). *Lactobacillus paracasei* CNCM-I 2116 (ST11) can alleviate skin inflammation *in vitro* by preventing TNF-α release, mast cell degranulation, vasodilation and oedema, thus accelerating the recovery of barrier function (Gueniche et al., 2010).

**TABLE 4:** Potential probiotics and prebiotics for psoriasis treatment.

Patients or mice	Probiotic intervention	Clinical outcome	References
Nc/Nga mice	Oral administration of poly-γ-glutamate	Suppressing the Th2-biased immune response and production of IL-17A	Chen et al., 2017
Female C57Bl/6 (2–4-month-old) mice	Oral treatment with 200 μl of live *L. casei* DN-114 001	Alleviated T cell-mediated skin inflammation without causing immune suppression, via mechanisms that include control of CD8+ effector T cells and involve regulatory CD4+ T cells	Vijayashankar and Raghunath, 2012
Specific pathogen-free (SPF) male C57BL/6J mice	(i) Probiotic (*B. animalis* subsp. *lactis* and *L. paracasei* subsp. *paracasei* DSM 46331), (ii) Prebiotic (oat β-glucan) (iii) Synbiotic (a mixture of i and ii) treatments for 12 weeks	Restored caecal levels of acetate, propionate, and butyrate; reduced body weight and alleviated features of metabolic complications	Ke et al., 2019
Male C57BL/6J mice at 7 weeks of age	A combination of *L. mali* APS1 and dieting	Accelerated body weight loss, reduced caloric intake, and lowered fat mass; ameliorated hepatic steatosis; affected fecal SCFA excretion and expression of satiety hormones	Chen Y. T. et al., 2018

Data on probiotic supplementation in psoriasis treatment are limited, but the pathogeneses of psoriasis and obesity have shown certain overlapping genetic and environmental factors as well as immune pathways. Th17 cells and their cytokines play an important role in psoriasis progression and the pathophysiology of obesity, indicating that probiotics, which are effective for obesity treatment, may also be effective for psoriasis treatment. The gut microbiota of obese individuals is less diverse than that of non-obese individuals, with a reduction in Gram-negative bacteria, specifically members of *Bacteroidetes*, and an increase in Gram-positive *Firmicutes* (Ley et al., 2006; Turnbaugh et al., 2009), which can also be observed in psoriatic patients (Ley et al., 2006; Turnbaugh et al., 2009). Specific strains belonging to *Lactobacillus* [*L. casei* strain Shirota (LAB13), *L. gasseri*, *L. rhamnosus*, and *L. plantarum*, among others] and *Bifidobacterium* (mainly *B. infantis*, *B. longum*, and *B. breve B3*) species, as well as other microorganisms, including *Pediococcus pentosaceus* LP28, *Bacteroides uniformis* CECT 7771, *Akk. muciniphila*, and *Saccharomyces boulardii* Biocodex, have shown anti-obesogenic effects in animals (Ukibe et al., 2015; Alard et al., 2016; Li et al., 2016; Bubnov et al., 2017; Shin et al., 2018; Avolio et al., 2019). Among these, the effects of *L. casei* and *B. infantis* strains have been confirmed. Notably, there is no evidence of the long-term effects of probiotics either as human food supplements or as adjunctive therapy. Therefore, the safety of these bacteria needs to be rigorously assessed before application in the treatment of various diseases.

## Conclusion and Perspectives

This systematic review revealed that microbiome alterations, such as abnormal colonization by *C. albicans* and *S. aureus*, may act as potential pathogenic factors for psoriasis. The skin and intestinal microbes of patients with psoriasis were significantly different from those of healthy subjects. However, due to the lack of standardized protocols, the microbial communities of patients with psoriasis were hardly comparable among the various study results, and it was difficult to confidently identify changes in the microbial communities associated with the psoriasis disease status. More rigorous research or robust statistical techniques might provide a credible answer. Microbiological interventions, including fecal transplants and probiotics, have been used in patients and mice with psoriasis, especially in patients who have not responded to multiple other therapies. However, the long-term effects of these interventions have not been reported and should be carefully evaluated in clinical applications, especially for younger patients. Obesity is closely related to psoriasis, and the two have a bidirectional effect. Understanding the role of microbes in the interaction of pathogenesis in two conditions may provide access to novel treatments.

## Author Contributions

LC developed the writing of the manuscript, figure, and tables. CP designed the review and contributed to proofreading and revising it with WZ. All the authors contributed to the article and approved the submitted version.

## Conflict of Interest

The authors declare that the research was conducted in the absence of any commercial or financial relationships that could be construed as a potential conflict of interest.
